# Quantifying daily rhythms with non-negative matrix factorization applied to mobile phone data

**DOI:** 10.1038/s41598-022-09273-y

**Published:** 2022-04-01

**Authors:** Talayeh Aledavood, Ilkka Kivimäki, Sune Lehmann, Jari Saramäki

**Affiliations:** 1 Department of Computer Science, Aalto University, Espoo, Finland; 2 Department of Applied Mathematics and Computer Science, Technical University of Denmark, Kongens Lyngby, Denmark; 3 Copenhagen Center for Social Data Science, University of Copenhagen, Copenhagen, Denmark

**Keywords:** Computational science, Machine learning

## Abstract

Human activities follow daily, weekly, and seasonal rhythms. The emergence of these rhythms is related to physiology and natural cycles as well as social constructs. The human body and its biological functions undergo near 24-h rhythms (circadian rhythms). While their frequencies are similar across people, their phases differ. In the chronobiology literature, people are categorized into morning-type, evening-type, and intermediate-type groups called *chronotypes* based on their tendency to sleep at different times of day. Typically, this typology builds on carefully designed questionnaires or manually crafted features of time series data on people’s activity. Here, we introduce a method where time-stamped data from smartphones are decomposed into components using non-negative matrix factorization. The method does not require any predetermined assumptions about the typical times of sleep or activity: the results are fully context-dependent and determined by the most prominent features of the activity data. We demonstrate our method by applying it to a dataset of mobile phone screen usage logs of 400 university students, collected over a year. We find four emergent temporal components: morning activity, night activity, evening activity and activity at noon. Individual behavior can be reduced to weights on these four components. We do not observe any clear categories of people based on the weights, but individuals are rather placed on a continuous spectrum according to the timings of their phone activities. High weights for the morning and night components strongly correlate with sleep and wake-up times. Our work points towards a data-driven way of characterizing people based on their full daily and weekly rhythms of activity and behavior, instead of only focusing on the timing of their sleeping periods.

## Introduction

Our lives are defined by rhythms of different frequencies: daily, weekly, seasonal, and annual, among others. The most prominent rhythms in our lives are rooted in the day-night cycle^1^. From biological cell functions to social activities and interactions, many aspects of human lives follow diurnal rhythms^2^. Sleep-wake cycles are regulated by circadian rhythms which are internal body processes^3^ as well as by external cues such as the amount of light and factors related to our social lives. Sleep is important to our health and well-being and it enables us to restore physically and mentally to pursue our daily activities^4^. Circadian rhythms have a similar near-24-hour length for all humans, but their phase (i.e., the relative timing of the circadian cycle) varies from one person to another which results in different sleeping hours^5^. There is a large body of literature that shows that people with late circadian rhythms are at risk of various physical and mental issues^6–8^. Therefore, it is crucial to measure and to understand these rhythms.

In chronobiology, people are commonly divided into three categories referred to as “chronotypes”^9^ according to the difference in the phase of circadian rhythms and their tendency to sleep at different hours. This tendency has a genetic dimension and is also affected by other external variables such as social norms and typical hours for working, sleeping, eating, and other social interactions. The way chronotypes are measured is either by asking people about their personal preferences (if they were to have no obligations) or by asking how their internal body clock is aligned with the day-night cycle^10^. The three widely-accepted chronotypes are morning type, evening type, and intermediate type^11^. Chronotypes are often measured with one of the various available questionnaires which have been developed for this purpose since the 1970s^11^. Like any other questionnaire, these questionnaires rely on a person’s description of their behavior rather than measuring the behavior in situ. It is also typical for studies that use chronotype questionnaires to draw lines at certain values of the continuous questionnaire outcomes to derive different categories. For example, one of the most well-known chronotype questionnaire, the Morningness–Eveningness Questionnaire (MEQ)^12^, was originally designed based on a cohort of adults with ages between 18 to 32 years old^11^. There are also chronotype questionnaires, such as the Munich ChronoType Questionnaire (MCTQ)^13^, which defines the output score of the questionnaire as a continuous variable.

In this work, instead of using questionnaires that rely on people’s memories, we measure their sleep through phone activity patterns based on digital traces that are recorded in an unobtrusive manner from mobile phones. Instead of matching our subjects’ activity patterns to another cohort, we use Non-negative Matrix Factorization (NMF)^14^ to automatically learn the dominant patterns in the data. We use this unsupervised method to reduce the dimensionality of the data and to extract four dominant rhythms within the population’s activity patterns. This approach minimizes the a priori assumptions on the sleep and activity hours of the study participants and allows these patterns to emerge from the data.

Our work is part of a rapidly growing field in which digital traces that people leave behind, such as data produced by mobile phones, are used as a proxy for human activity and to measure temporal patterns of their behavior^15–18^. These traces allow us to unobtrusively measure people’s behavioral patterns in their natural everyday settings. The continued evolution of mobile phones and in particular the ubiquity of smartphones have opened up possibilities for collecting high-resolution data from individuals and studying their behavior at a level of detail not previously possible^19^. In the past years, multiple studies have gathered detailed data from individuals to study and quantify their behavior^19–22^. More recently, high-resolution data from mobile phones have been used to study the sleeping and resting patterns of people^23–28^. Most of the prior work in this area focuses on inferring the hours of going to sleep and waking up from people’s activity on their phones. This is based on the assumption that many people (in particular those who are younger) use their phones frequently while they are awake, and often use the phone’s alarm to wake up in the morning^24,29^. Our work follows this assumption as well, but it pushes these analyses one step further by studying the rhythms of activity on the phone throughout the whole day instead of only detecting the sleep hours. Previous studies have shown the importance of studying behavioral and activity rhythms^30^ and how deviations from these rhythms could be an early sign of mental disorders^31^. It is therefore crucial to better understand the rhythms of sleep and activity. Our work introduces a new method to study temporal patterns of phone activity that can be applied to any temporal activity data from similar studies.

In this work, we demonstrate the use of our method by applying it to data from the Copenhagen Networks Study, which has collected data from around 1000 university students for over two years^19^. The majority of the students in this study were in the age range 19–21 and the demographics was male dominated^32^. In this study, participants were given identical phones, and, with their consent, data on their phone usage was collected and shared with researchers. The original objective of this study was to study social networks, mobility, and privacy^32^. Here, we use the timestamps of *screen-on events* where the phone screen turns on, recorded as part of the original data collection effort. The times and frequencies of these events allow us to measure the dominant activity and sleep rhythms in the studied population of students. We also study in detail how the component weights of the dominant rhythms correlate with one another within the population. Furthermore, we investigate their correlations with sleep and wake-time hours as well as the sleep duration.

Out of all study participants, over 800 students provided data for the study in the year 2014. For this analysis, we included data from 400 of these students after filtering out individuals with low activity levels (see section “Dataset and data pre-processing” for details on data pre-processing).

## Implementation and results

**Figure 1:**
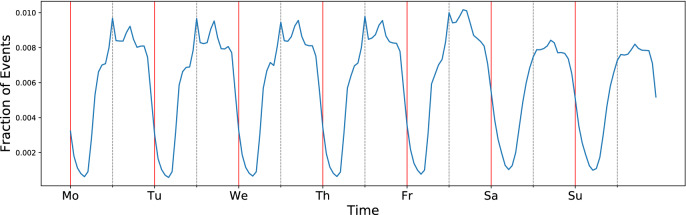
The normalized activity rhythm averaged over the whole student population ($N=400$). The horizontal axis spans one week, with time discretized into one-hour bins. The red lines mark midnight and the grey dashed lines indicate midday.

We use screen-on events as a proxy of the times when the person is active during the day (for details on data and preprocessing, see “Dataset and data pre-processing”). While screen-on events do not capture all types of activity, they are, for example, a good measure of when a person is awake and using the phone. By studying the temporal activity patterns of phone usage over a long period of time, we can form a picture of the behavioral patterns of the person. We aggregated each week’s screen-on event data for each person over the course of one year into one-hour bins (from Monday morning to Sunday night), so that each of the $7\times 24=168$ bins contains the number of screen-on events for that hour. These time series are then normalized for each person so that they represent the fraction of screen-on events for each hour of the week. The population average of these *activity rhythms* for all the 400 individuals is shown in Fig. 1. The average rhythm shows clear daily periodicity with a lower activity level during weekends, reflecting the common pattern in individual rhythms. The lowest levels of activity coincides with night times.

**Figure 2:**
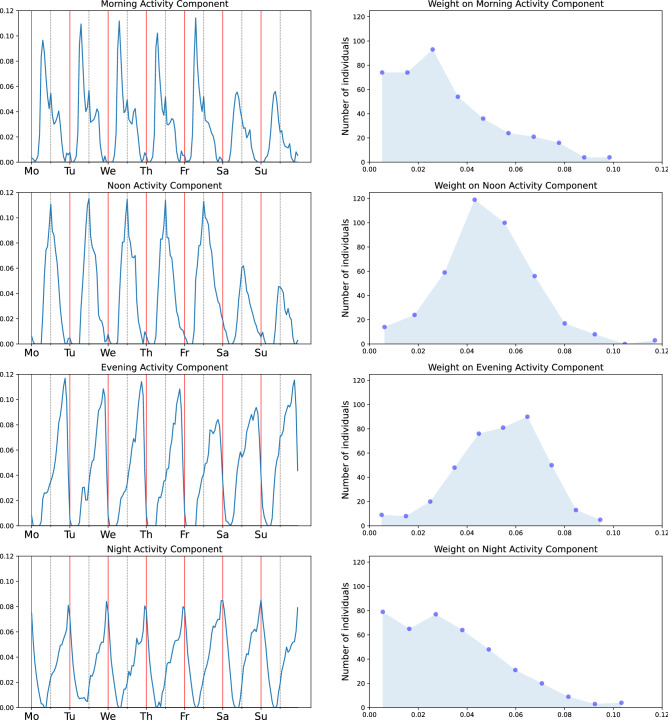
Left: the four activity rhythm components from NMF; each row of the figure corresponds to one component. Right: the histograms of the weights of each of the components in the student population, showing how many students have a given weight. The components are purely data-driven and detected by the NMF method without using any external information on the daily rhythms.

We used NMF as an unsupervised method to extract the four main components of the activity rhythms of the 400 students included in the analysis (for details on the choice of method and number of components see section “The rationale for choosing NMF and the number of components”). The four NMF components are displayed in Fig. 2. The components follow a daily periodicity, peaking at different hours of day. The timings of the four peaks are associated with morning, noon, evening, and night.

Of the four components, the morning and noon components show lower levels of activity on weekends than on weekdays. The evening activity component is lower on Friday and Saturday evenings, and the night activity component remains approximately similar on all days of the week. As components other than nighttime activity are lower in the weekends, there is a shift in the activity patterns of people towards later hours in the weekends, which is consistent with the typical behavior of adults who often go to bed earlier on weekdays and stay up later and catch up on sleep at the end of the week^13^.

Figure 2, right column, shows the distributions of weights on the four components in the student population. The distributions of the weights on the noon and evening components resemble a normal distribution centered around a mean value. However, the distributions of the weights on the morning and night components are skewed. This means that while the vast majority of students has low weights on these two components, there is a small fraction of students with larger weights.

**Figure 3:**
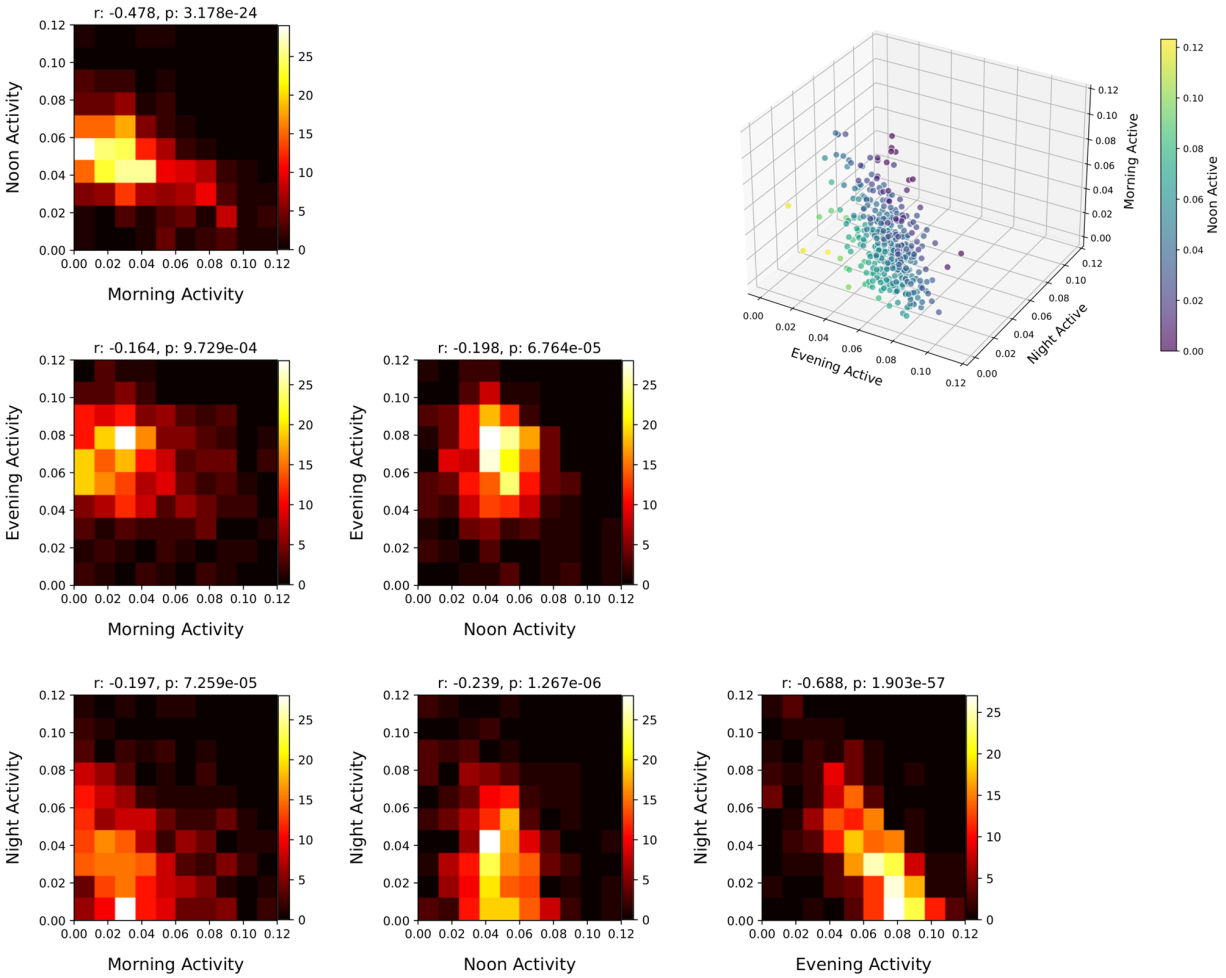
Correlation between weight on one component vs. the other components. The color depicts the number of people with values within each square. On top of each panel, the value of the Pearson correlation coefficient (left) and the corresponding p-value (right) are shown. The highest (anti)-correlations are between the Evening and Night components (− 0.688) and Morning and Noon components (− 0.478), meaning that people tend to be more active in either of each the two components for each pair. In the top right 3D plot, each dot represents one person so that the weights on three components (morning, evening and night) are the coordinates in the 3D space and the weight on the fourth component (noon) is represented by the color of the dot. Despite the existence of some outliers, people tend to form a continuous spectrum within this space. High values of weights for the Evening activity coincide with low weights on all other dimensions. The sum of the four weights for each person is normalized to unity.

At the level of individuals, the weights of the four NMF components are not independent. The correlations between the component weights are explored in Fig. 3, where the heatmaps represent the numbers of students with component weights within specified intervals. While the correlation between some weights is low (morning and evening, morning and night, noon and evening), some of the components are clearly negatively correlated. For example, the Pearson correlation coefficients for the weights on the night and evening components is − 0.69, and therefore people do not typically have high weights on both of these components simultaneously. Fig. 3, top right, shows the whole population in terms of their four component weights (three axes and symbol color). In this plot, we see that individuals cannot be easily grouped into separate groups based on their phone activity rhythms, and their weights on the four components form a continuous spectrum. This spectrum has, however, an elongated form (rather than a symmetrical cloud) and shows that high weight of the evening component coincides with low values on all other components.

**Figure 4:**
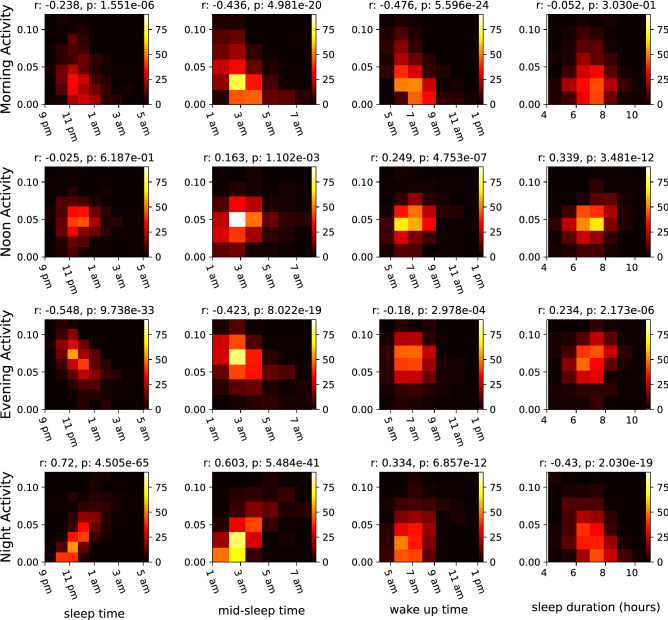
Correlations of different NMF components with four different parameters, from left to right: sleep time, mid-sleep time, wake-up time and sleep duration.

Sleep is a substantial part of people’s days and therefore one of the main determinants of how the daily rhythm of a person looks. Long sleeping episodes are visible in the daily rhythms as long periods of inactivity. These periods of inactivity are reflected in the NMF components and their weights. To investigate this association, we first directly calculate the most frequent going-to-sleep time, wake-up time, mid-sleep time, and sleep duration from the phone activity data (rather than using the NMF components or the weekly averages). We then examine the correlation of these variables with weights on the different components (see Fig. 4). For details on how these variables are estimated from the data, see section “Calculating the typical sleep and wake-up times”.

Sleep time shows a moderate anti-correlation with the weight on the evening activity and a high correlation with the weight on the night activity component (see Table 1). This implies that going to sleep later is associated with higher activity on the phone on late hours of the day. This finding is non-trivial because staying up later does not necessarily mean that the person has to be more active on the phone. For calculating sleep times, even one data point at a late hour (e.g. while setting up the alarm before going to bed) is sufficient for our algorithm to determine a late sleep time. However, for a high weight on the night activity component, the person has to have many data points (screen-on activity) at late hours. This also implies that those who have a high evening activity tend to be early sleepers, while those with high night activity levels are late sleepers. Similarly, the wake-up time shows a moderate anti-correlation with the morning activity component, meaning that an earlier wake-up time is associated with higher activity level on the phone in the morning. The third and fourth columns in Fig. 4 show the mid-sleep point and sleep duration vs weight on different components. The mid-sleep time is the middle point between sleep time and wake-up time, and therefore it can be the same for two people with different sleep durations. The mid-sleep time shows moderate to high (anti-)correlations with the morning activity, evening activity and night activity components. Sleep duration shows a moderate anti-correlation with the night activity component and a moderate correlation with the noon activity component.

**Table 1:** Pearson correlation coefficients for the four NMF components and sleep and activity parameters: sleep time, wake-up time, mid-sleep time, and sleep duration.

		Sleep time	Mid-sleep time	Wake-up time	Sleep duration
Morning activity	r	− 0.238	− 0.436	− 0.476	− 0.052
	p-value	1.551e−06	4.981e−20	5.596e−24	3.030e−01
Noon activity	r	− 0.025	0.163	0.249	0.339
	p-value	6.187e−01	1.102e−03	4.753e−07	3.481e−12
Evening activity	r	− 0.548	− 0.423	− 0.18	0.234
	p-value	9.738e−33	8.022e−19	2.978e−04	2.173e−06
Night activity	r	0.72	0.603	0.334	− 0.43
	p-value	4.505e−65	5.484e−41	6.857e−12	2.030e−19

## Methods

### Dataset and data pre-processing

The dataset used in this study comes from a large data collection effort^19^ that followed a large number of volunteer students on a campus in Copenhagen, using mobile phones equipped with a tracking app. To analyze the activity of the study participants on their phone, we import data on the status of the phone screen in the form of comma-separated files where each row represents one instance of a phone screen (belonging to one of the students) turning on or off. The data row contains the identifier (ID) of the study participant, the event type (screen on or off), and the time of the event. We then apply extra filters to this data. We keep the data from weeks 2–51 of the year 2014, discarding data from weeks 1 and 52 because the first week partially lies in 2013 and on week 52 the Christmas holidays lead to atypical temporal rhythms. There were in total $N=804$ students that used their study phones during this year. We excluded study participants who did not use their phone actively or did not use it at all for part of the year. The inclusion criteria were: (1) the person should have used the phone on $80\%$ of the days, (2) during weeks 2–51, the participant should on average have 280 screen-on and screen-off events. The final number of study participants kept for further analysis was $N=400$. From here on, we call the output of this pre-processing stage the “phone activity data”. The same pre-processing steps as described above were also used in a previous study based on this dataset^25^.

### Using NMF to extract the dominant components of the weekly activity patterns

In order to extract common temporal patterns from the phone activity data, we use non-negative matrix factorization (NMF). NMF yields a (small) number of typical patterns for a dataset such that each column (time series) of the original data can be approximated as a weighted sum of those typical patterns. The constraint that the components must be non-negative makes the interpretation of the decomposition more intuitive than, e.g., for principal component analysis. Empirically, it has been found that NMF tends to produce components that correspond to individual parts of a system—when faces are decomposed, the components become eyes, noses, mouths, and moustaches^33^.

For the NMF decomposition, we store our weekly activity data (the weekly time-series) in an $N \times M$ matrix $\mathbf {X}$, where *N* is the number of data vectors (in our case, the number of individuals studied) and *M* their dimensionality (here, the number of hours in a week). In NMF, the matrix $\mathbf {X}$ is approximated as a product of two lower-dimensional matrices as

1 $$\begin{aligned} \mathbf {X} \approx \mathbf {WH}^{\mathbf{T}}, \end{aligned}$$

where $\mathbf {H}$ is an $M \times K$ and $\mathbf {W}$ an $N \times K$ matrix, and *K* is the number of components sought from the data. The components are the *K* column vectors of matrix $\mathbf {H}$ (i.e., the rows of $\mathbf {H}^{\mathbf{T}}$). These components are vectors in the original data space, and so in our case they represent weekly activity time series. Each original vector of data is now approximated as a superposition of these *K* component vectors. The contribution of each component to this approximation is given by the elements of the matrix $\mathbf {W}$. For example, the first data vector, $\mathbf {x}_1$, i.e. the first row of the matrix $\mathbf {X}$, is approximated in NMF as $\mathbf {x}_1 = w_{11} \mathbf {h}_1^{\mathbf{T}} + w_{12} \mathbf {h}_2^{\mathbf{T}} + \ldots +w_{1K} \mathbf {h}_K^{\mathbf{T}},$ where $\mathbf {h}_i^{\mathbf{T}}$ are the rows of matrix $\mathbf {H}^{\mathbf{T}}$.

The approximation of Eq. (1) is achieved by minimizing the error between the actual data matrix and the factorization. Any meaningful error function can be used, and we chose the commonly used squared Frobenius distance which generalizes the Euclidean distance from vectors to matrices:

2 $$\begin{aligned} E = \tfrac{1}{2}\Vert \mathbf {X} - \mathbf {W}\mathbf {H}^{\mathbf{T}} \Vert _{\mathrm {Fro}}^2 = \tfrac{1}{2}\sum _{i,j} \left( x_{ij} -\sum _k w_{ik} h_{jk}\right) ^2. \end{aligned}$$

For computing the NMF, we used the scikit-learn Python package^34^, which contains an off-the-shelf implementation of the Hierarchical Alternative Least Squares (HALS) algorithm for minimizing the error of Eq. (2)^35^. The implementation is stochastic, and therefore we always ran the algorithm with 1000 different random seeds, picking the run that resulted in the smallest error.

### The rationale for choosing NMF and the number of components

**Figure 5:**
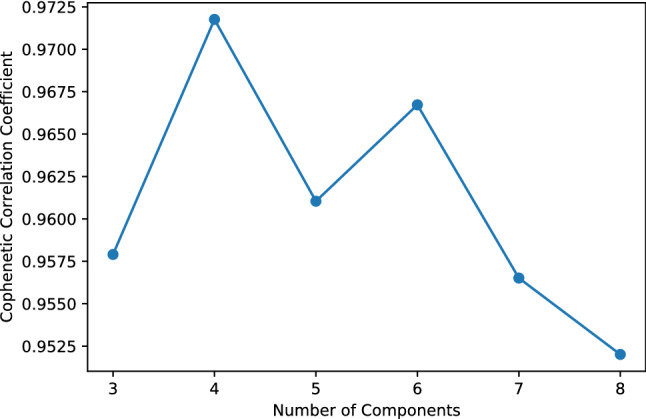
Cophenetic correlation coefficient versus the number of components.

The extraction of the dominant rhythms of the system can be approached with several different tools, including principal and independent component analyses, factor analysis, topic modeling, as well as some functional data analysis methods. We experimented with some of these methods, but found the results obtained with NMF most informative (see the Supplementary Information section). Our main rationale for choosing NMF over the other methods was its interpretability, e.g. compared to component analysis methods which do not have the non-negativity constraint, and its conceptual simplicity over more involved methods such as topic modeling and functional data analysis methods. The use of NMF for temporal data has also proven to be successful in past studies^36,37^. Also, the frequency of screen-on events is always non-negative, which further makes NMF a natural choice. For the optimal number of components, we started with three components which is the number of commonly used chronotypes. We then investigated the optimal number of components using the cophenetic correlation coefficient, which is a measure of finding optimal number of components in NMF based on stability of components in different runs of the algorithm. We found that the cophenetic correlation coefficient is maximized for four components (see Fig. 5). For calculating the cophenetic correlation coefficient we used Nimfa, a Python library for NMF^38^.

**Figure 6:**
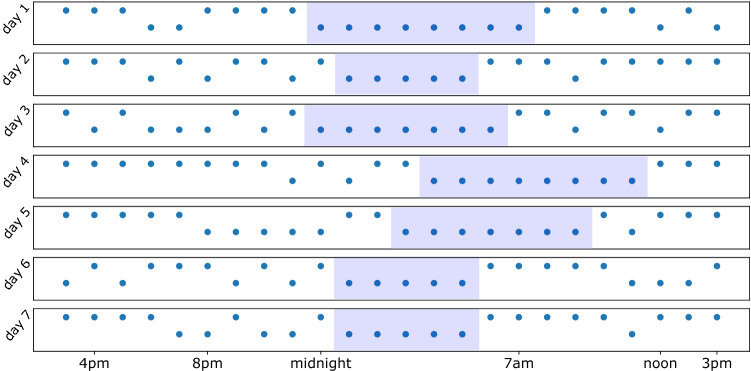
This figure shows presences of screen-on events in different hours and different days (each row is one day of data for 7 sample days) from one person in the study. The higher dots show presence of phone activity in that hour and lower dots show the lack of it. We find the longest period of of inactivity within the 24 h (area filled with blue). These hours are marked as sleeping times. The first hour of inactivity is recognized as (going to) “sleep time” and the first hour right after of the period of inactivity is determined to be the “wake-up time”. This processes repeated for all days for each person.

### Calculating the typical sleep and wake-up times

For each 24-h period and for each person, we assume that the longest period of inactivity on the phone is when they are asleep. Using this assumption, we then estimate their sleep-and wake-up times for every day in the data (350 days in total) for inferring typical sleeping times^39,40^. Figure 6 displays the estimated periods of sleep for one person in the study, over a period of 7 days out of the total of 350 days. For calculating the common sleep and wake-up times for each person and each day, we find the longest period of inactivity and pick the first hour of the longest period of inactivity as the beginning of the sleep time. Similarly, we take the first hour of activity after the longest period of inactivity as the wake-up time.

From the sleep and wake-up time, we calculate the mid-sleep time. Then, from the 350 sleep, wake-up, and mid-sleep times for each person, we compute the mode of each of the three distributions and consider the modes their most common sleep, wake-up, and mid-sleep times.

The rationale of using the mode of the distributions is to reduce the effect of outlier nights. For calculating the typical sleep duration for each person, we simply compute average number of the hours of inactivity. This analysis leads us to one value for each of the four variables (typical sleep, wake-up, mid-sleep time, and sleep duration) for each of the 400 students included in this analysis.

## Discussion

In this work, we introduce a method for studying the rhythms of sleep and activity by applying non-negative matrix factorization (NMF) to time-stamped smartphone data. This method is entirely data-driven and requires no other inputs than the phone activity time series for each subject (in our case, time series of screen-on events).

Applying the method to a population of 400 university students, we see clear daily and weekly rhythms, with low activity during the night and differences between weekday and weekend patterns. The application of NMF yields four meaningful components with activity peaks at different hours of the day (morning, noon, evening and night components). Each student’s activity rhythm can be characterized with a superposition of these four components with different weights. The analysis of the weights reveals individual variation, which is consistent with findings from chronobiology studies: different people tend to sleep at different hours of the day and the times when they are most active or alert also vary. We also observe that in the population, the component weights form a continuous spectrum and do not exhibit clear-cut clusters.

An important feature of the NMF method is that it does not require a reference population and that it is entirely agnostic to what would be culturally considered as “late” or “early”: the components are purely determined from the data at hand. While it is well known that there are individual differences in the endogenous phase of the circadian rhythm, exogenous factors can also play a major role in determining the sleep and activity patterns of people. While some chronotype questionnaires try to capture these external factors, they still depend on data from a pool of people to determine the association between chronotype and questionnaire’s scores. With NMF, we reduce each person’s rhythm to weights on the extracted components. This way we can measure the typical timings of a person’s sleep and phone activity without needing a different population for comparison and for determining who should belong to each chronotype category. As an example, the high weight of an individual’s morning activity is to be interpreted as relative to the population at hand, which removes the risk of cultural and environmental biases in determining what makes someone a morning person.

An obvious limitation of this method is that since it builds on analyzing mobile-phone activity, it can only be applied to data on individuals who use their phones actively. When it comes to estimating patterns of sleep, whether this limitation gives rise to biases at the population level depends on whether those patterns correlate with the lack of phone use, or are distributed among non-users similarly as in the rest of the population. This problem is, however, generally similar for all studies that build on collecting data from electronic devices.

It would be very interesting to investigate whether similar components emerge when studying populations with different demographics and lifestyles, and different time frames (e.g. variations between seasons). Even though our method is not directly designed to detect changes in the temporal patterns of phone usage, future work can also expand on this analysis and quantify changes in phone activity rhythms over time or at specific points in time. It would also be informative to use a dataset where both phone activity data and chronotype questionnaires are available. This would allow comparing questionnaire categories with those derived by using NMF together with some clustering method applied to the component weights. In addition, the same method could be applied to other types of data collected from phones and other wearables which have (at least) an hourly time resolution. This would allow us to study behavioral patterns related to other types of activity and compare them across different data streams. For example, in a previous work, we showed that individuals tend to have persistent daily rhythms for communication across different channels (calls and text messages) but the shapes of these rhythms and peak hours differ^41^. By studying the rhythms of different data streams, we could for example investigate how the rhythms of social activity differ from those of physical activity.

Some of the previous questionnaire-based works on chronotypes have pointed out that using thresholds in these questionnaires for separating different groups is rather meaningless^13^, and it has been suggested that there is a wide and continuous spectrum of individual types. The results of our data-driven method applied to a student population are in line with this—we see no support for the use of strictly categorical variables such as chronotypes for describing people’s activity and sleep habits.

Considering the chronotype as a continuous variable instead might improve the results of studies related to adverse health impacts of late phase of the circadian rhythms. Given the work presented here, we believe that there is a need to rethink how to quantify temporal patterns in human behavior and to possibly extend the concept of chronotype so that it takes the nuances of the activity level during non-sleep periods better into account.

## Supplementary Information

Supplementary Figures.

Supplementary Information.
