# Supplementary material for: Quantifying daily rhythms with non-negative matrix factorization applied to mobile phone data
Source: Sci Rep. 2022 Apr 1;12:5544. doi: 10.1038/s41598-022-09273-y (PMC8975853; doi:10.1038/s41598-022-09273-y)
Supplement: Supplementary file 1 — Supplementary Figures. [file 41598_2022_9273_MOESM1_ESM.pdf]

Supplementary information

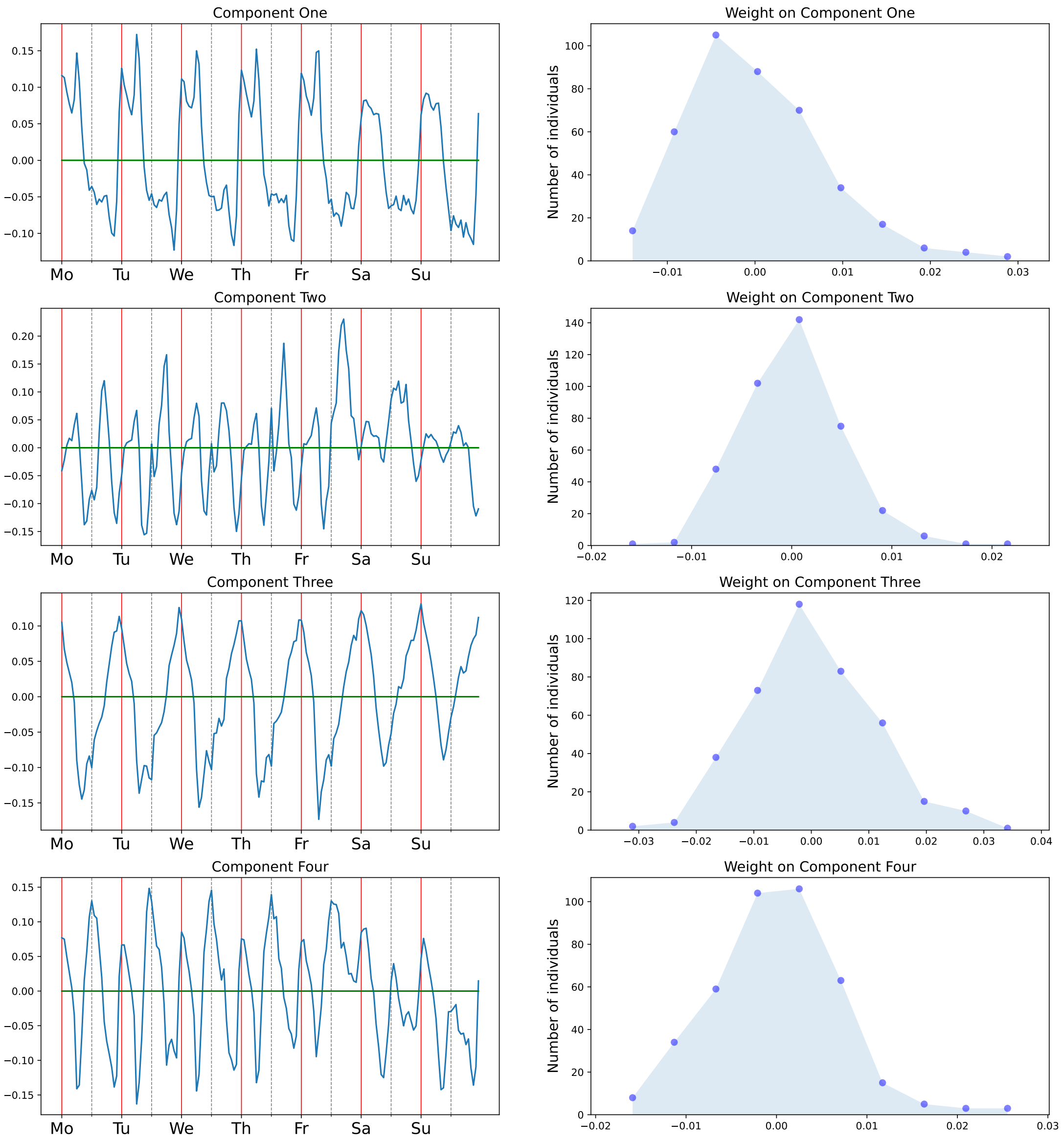

Supplementary Figure 1. Left: The four components derived using principal component analysis (PCA). Right: The histograms on the right depict how many people have a certain weight; each panel is associated with one of the four PCA components. Unlike NMF, the PCA components can be negative as seen in the left panel, and this makes their interpretability more difficult.

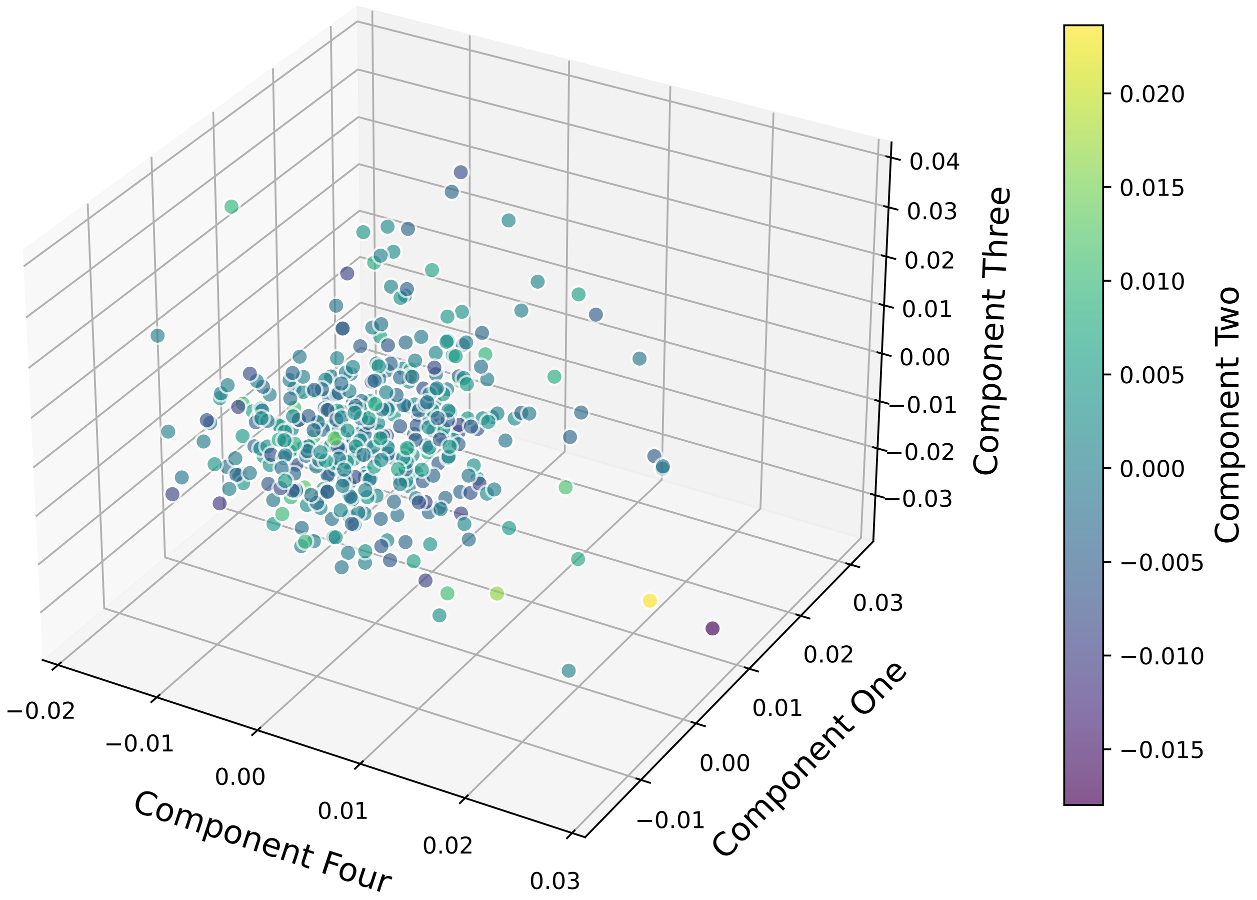

Supplementary Figure 2. In this figure, each dot represents one person so that the weights on three PCA components are the coordinates in the 3D space and the weight on the fourth component is represented by the color of the dot. Similar to NMF, no clear clusters emerge using this method.

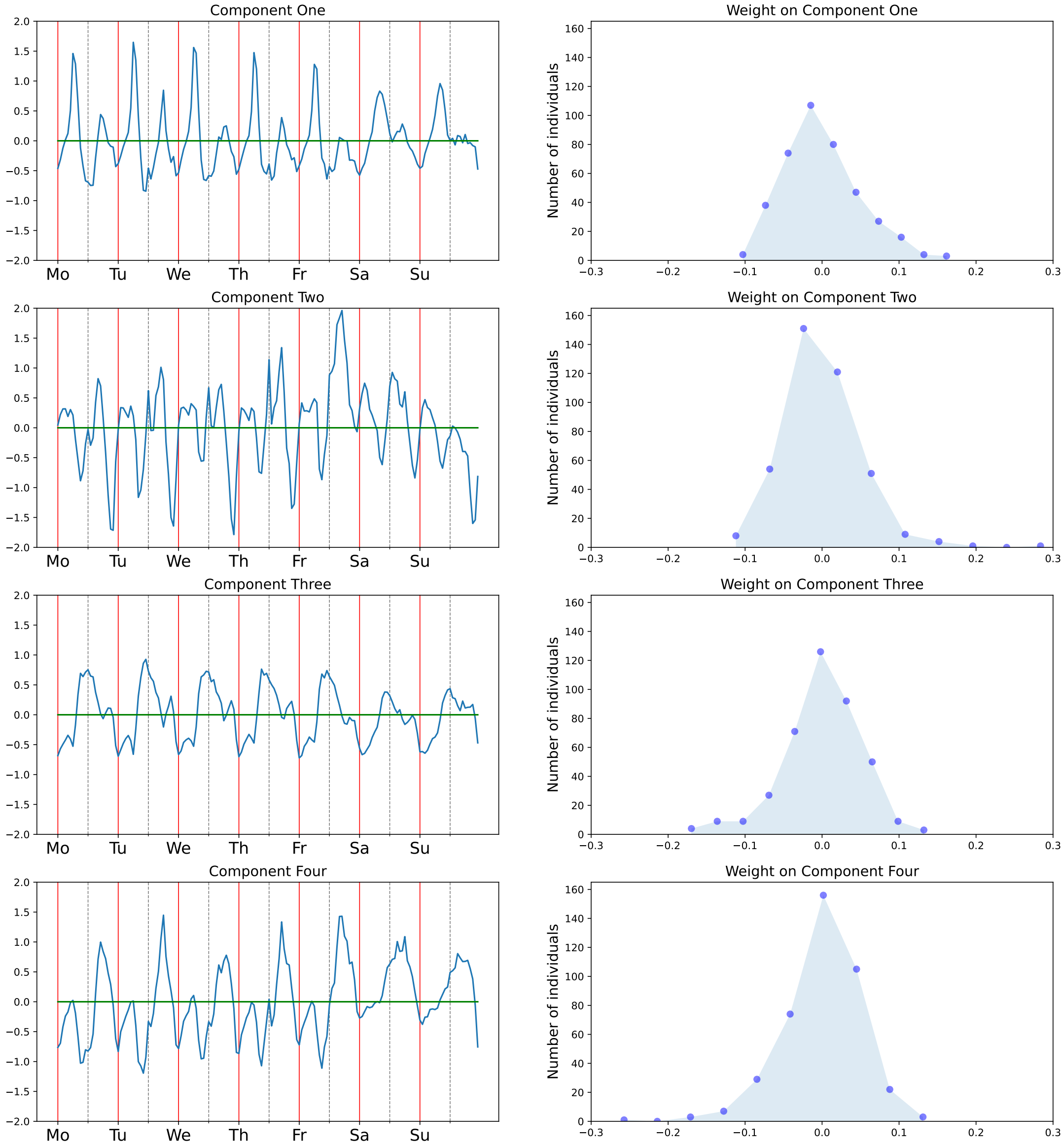

Supplementary Figure 3. Left: The four components derived using independent component analysis (ICA). Right: The histograms on the right, depict how many people have a certain weight; each panel is associated with one of the four ICA components. Unlike NMF, and similar to PCA, the ICA components can be negative as seen in the left panel, and this makes their interpretability more difficult.

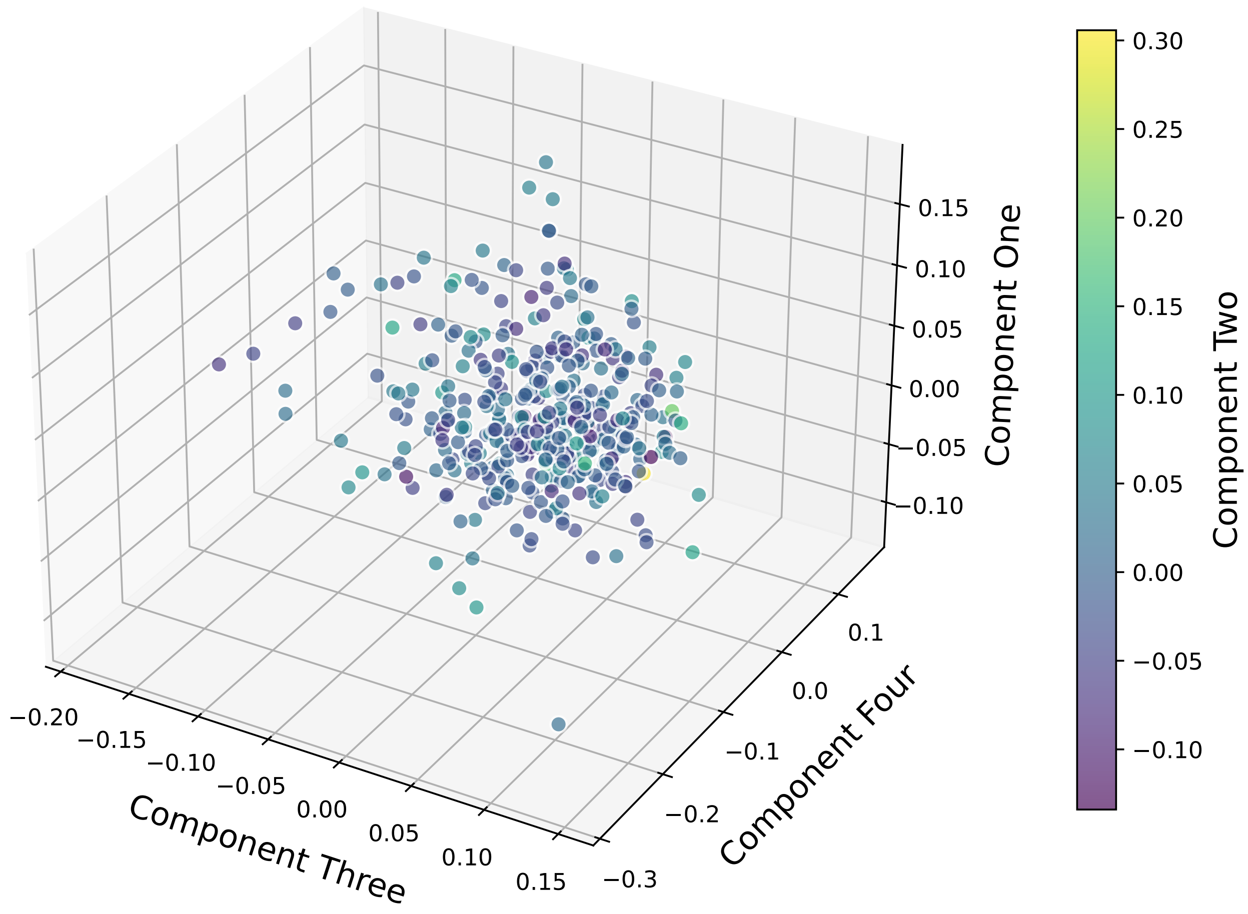

Supplementary Figure 4. In this figure, each dot represents one person so that the weights on three ICA components are the coordinates in the 3D space and the weight on the fourth component is represented by the color of the dot. Similar to NMF, no clear clusters emerge using this method.
